# Translation of Methodology Used In Human Myocardial Imaging to a Sheep Model of Acute Myocardial Infarction

**Published:** 2013

**Authors:** Elizabeth A Bailey, Dale L Bailey, Stephen Hunyor, Leigh Ladd, George J Bautovich

**Affiliations:** 1 Department of Nuclear Medicine, Royal North Shore Hospital, Australia; 2 Department of Cardiology, Royal North Shore Hospital, Australia; 3 Discipline of Medical Radiation Sciences, University of Sydney, Australia; 4 Sydney Medical School, University of Sydney, Australia; 5 Cardiac Technology Centre, North Shore Heart Research Group, Kolling Institute, Australia; 6 Charles Sturt University, Wagga Wagga, Australia

**Keywords:** Myocardial perfusion Imaging, SPECT/CT, Ovine model, Mesenchymal stem Cells

## Abstract

**Introduction:**

Pre-clinical investigation of stem cells for repairing damaged myocardium predominantly uses rodents, however large animals have cardiac circulation closely resembling the human heart. The aim of this study was to evaluate whether SPECT/CT myocardial perfusion imaging (MPI) could be used for assessing sheep myocardium following an acute myocardial infarction (MI) and response to intervention.

**Methods:**

Eighteen sheep were enrolled in a pilot study to evaluate [^99m^Tc]-sestamibi MPI at baseline, post-MI and after therapy. Modifications to the standard MPI protocols were developed. All data was reconstructed with OSEM using CT-derived attenuation and scatter correction. Standard analyses were performed and inter-observer agreement was measured using Kappa (κ). Power determined the sample sizes needed to show statistically significant changes due to intervention.

**Results:**

Ten sheep completed the full protocol. Data processed was performed with pre-existing hardware and software used in human MPI scanning. No improvement in perfusion was seen in the control group, however improvements of 15%-35% were seen after intra-myocardial stem cell administration. Inter-observer agreement was excellent (К=0.89). Using a target power of 0.9, 28 sheep were required to detect a 10-12% change in perfusion.

**Conclusion:**

This study demonstrates the suitability of large animal models for imaging with standard MPI protocols and its feasibility with a manageable number of animals. These protocols could be translated into humans to study the efficacy of stem cell therapy in heart regeneration and repair.

## Introduction

Advances in developmental and cell biology have created great interest in the potential of stem cell therapy to repair and regenerate the damaged heart, however, substantial challenges remain before this becomes an effective and safe therapeutic option (1). One of the major issues is the discrepancy between positive outcomes from stem cell therapy in rodent models following experimental myocardial infarction (MI) and the results from clinical trials performed in humans (2-5). While rodent studies show functional improvements of 15-20% in heart pumping capacity, a meta-analysis of human trials only showed a small transient (~4%) improvement in left ventricular ejection fraction (LVEF), with one study showing no benefit(6). Complexities arise partly from the use of a variety of stem cells including embryonic (ESC) and adult stem cells (ASC) derived from several tissues such as bone marrow, induced pluripotent stem cells (iPSC) and resident cardiac stem or progenitor cells (7-9).

The up-scaling from basic science in rodents to clinical trials in humans often encounters a “translational gap” which can be overcome by the use of an intermediate stage using large animals. Functional and cellular consequences of myocardial infarction and response to therapy can be readily studied in sheep and pigs with techniques directly applicable to human subjects due to similarities in heart size, anatomy, coronary supply and molecular machinery(10). One such example is the use of stem cells to repair damaged myocardium where controversy abounds as to the appropriate method of administration of the cells i.e., systemic, intra-arterial or directly into the myocardium.

Many imaging studies with mesenchymal stem cells (MSCs) have attempted to trace the fate of the implanted cells using a label suitable for imaging. Studies on rodents using direct injection of [^111^In]-labelled bone marrow-derived MSCs into the affected area of heart muscle to assess stem cell therapies have presented difficulties due to the small heart size (11). Imaging the fate of transplanted cells using labelling techniques with radionuclides, paramagnetic iron oxide with MRI, or optical probes are problematic (11, 12). The relatively short half-life of some radiotracers may preclude long-term tracking and the radiation effects on cell viability have not been determined, making such studies largely unsuccessful and challenging in humans (13).

There are no published studies using an ovine (sheep) model to study myocardial function using SPECT/CT. We therefore set out to investigate the potential to image the myocardium before and after stem cell transplantation using readily available myocardial perfusion imaging (MPI) techniques. The aim of the study was to evaluate the potential for SPECT/CT and diagnostic tools routinely used in the clinical setting to assess myocardial injury and potential recovery following stem cell transplantation in a sheep model of acute myocardial infarction. If successful, these approaches could be widely employed to more rapidly facilitate translation from the animal model into man.

## Methods

A pilot study approved by the local Institutional Animal Care and Ethics Committee (RNHS/UTS 0504-018A) was undertaken to study eighteen Border Leicester/Suffolk-cross female sheep using MPI SPECT/CT at (i) baseline, (ii) following myocardial infarction, and (iii) after therapeutic intervention.

### 

#### Imaging Parameters and Protocols

**Figure 1:**
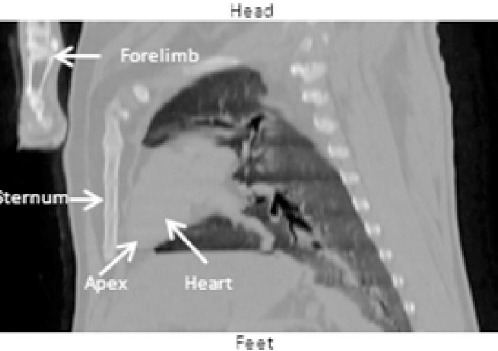
Coronal projection from the chest CT performed on sheep 12 showing the orientation of the heart in the human standing position. Alignment of the sheep heart is mirrored as compared to the human orientation.

All studies were acquired using a hybrid SPECT/CT system consisting of a SKYLight gamma camera (Philips, Milpitas, USA) and a PQ5000 CT scanner (Picker Corp, Cleveland, USA) (14). The sheep were positioned in the right decubitus position with the limbs folded and secured against the torso. In this position the heart has an orientation with respect to the detector above the animal, similar to a left anterior oblique in man with the apex of the heart being caudal (equivalent to inferior) and medial, and the base of the heart more cranial (equivalent to superior) and to the left, as seen in Figure 1.

A resting MPI study was commenced 15 minutes after injection of 1.5 GBq [^99m^Tc]-Sestamibi (Cardiolite^®^, Lantheus Medical Imaging, Massachusetts, USA) to assess myocardial perfusion and function. The acquisition consisted of a gated MPI SPECT study using a 128×128 matrix with 16 time bins at 15 seconds per projection for 120 projections (60 per detector, 3° radial increments) using a high-resolution collimator (VXGP), followed by a CT scan using a fixed beam current of 100 mAs, a tube voltage of 120 kVp and a pitch of 1 giving a slice thickness of 4 mm. After two days, an MI was induced using a 60 minute balloon occlusion (5F Judkins catheter, MayoHealthcare) of the dominant branch of the paraconal artery (equivalent to the left anterior descending artery in man) using a carotid artery cut-down approach followed by reperfusion. Between 5 and 7 days after MI, a repeat MPI study was performed to confirm the presence, location, extent, size and severity of the defect. If a significant or measurable defect was identified, then the sheep were allocated to one of three arms of the study with the imaging team remaining blinded to the final treatment arm:

- Group 1: the control sheep were given 1 mL of a placebo medium (stem cell suspension) injected directly into the myocardium;
- Group 2: MSCs (10 × 10^6^ cells) in suspension were infused over 1 minute via intra-arterial catheter (IC) into the left coronary artery with the cells most commonly injected into the arteries supplying the border zones of the MI (15);
- Group 3: MSCs (10 × 10^6^ cells) were delivered by intra-myocardial (IM) methods using 10 injections of 0.1 mL directly into the peri-infarct zone of the myocardium either under direct vision at thoracotomy or epicardially via cardiac catheter into the injured “border zone”(16).

Five to six weeks following the intervention the standard MPI imaging protocol was repeated as outlined above.

#### Anaesthesia Protocol

The sheep were fasted of solids but allowed water *ad libitum* the night before anaesthesia to decrease the risk of aspirating stomach contents and to avoid interference with cardiac imaging. The cephalic vein in the neck was cannulated for the anaesthesia. For the myocardial perfusion studies, light anaesthesia was induced with Alfaxalone (Alfaxan, Jurox, Australia) at a dose of 1 to 1.5 mg/kg I.V. and then maintained with isoflurane in oxygen titrated to maintain effect while respiration was controlled by intermittent positive pressure ventilation. Pupil size, jaw tone, movement, ECG and blood pressure were used to monitor the level of anaesthesia. Surgery was performed by an experienced cardiovascular surgeon to minimise tissue damage and any procedure related complications. Post-operatively the sheep were treated with Buprenorphine and Carprofen (a non-steroidal anti-inflammatory) for analgesia and were continuously monitored until standing, at which point respiration and heart rate stabilised and normal eating and drinking behaviour resumed.

#### Image Reconstruction of Myocardial Perfusion Images

Studies were reconstructed using two methods: Filtered Back-Projection (FBP) (200% zoom and 2D pre-filtering with Butterworth filter at an order of 10 and cut-off of 1.0 cycle/cm followed by FBP using a ramp filter) and an iterative reconstruction using the Ordered Subset Expectation-Maximisation (OSEM) algorithm (17) using 8 subsets of the projection data and 4 iterations. After reconstruction the data were filtered with a 3D Butterworth filter (order of 1.2 and cut-off of 0.8 cycles/pixel). Due to the different anatomical orientation of the sheep heart compared to the human heart, the studies were rotated +270° during reconstruction and then re-oriented -180° in the X-Y direction and -120° in the Y-Z plane to generate conventional cardiac oblique short axis slices.

Attenuation and scatter correction were performed using a CT-derived attenuation map that was generated by converting the CT Hounsfield units to the linear attenuation coefficients (μ) for ^99m^Tc (18). A transmission-dependent scatter correction (TDSC) method using the CT data was used to correct for scatter in the projection data (19). This then allowed for quantitative analysis of the data. The resultant transverse files were resampled into a 64×64 matrix (a requirement of the analysis software) with the heart centred in the field of view and reviewed qualitatively using the Cedars-Sinai QPS cardiac software review package (20). Investigators analysing the studies were blinded to the treatment group.

#### Data Analysis

Polar maps of myocardial perfusion were generated using the QPS package with a 20 segment scoring system of relative perfusion. These relative perfusion scores were used to calculate a normalised perfusion index for each segment on both the immediate post-MI images and post-therapy images. Perfusion recovery (or mean change) as a percentage of baseline was then calculated for each study. LVEF, end diastolic volume (EDV) and end systolic volume (ESV) were calculated from the gated MPI images at baseline, post-MI and post-therapy to assess LV functional change using the 4DMSPECT cardiac software review package (21).

The data were also qualitatively reviewed independently by two experienced nuclear medicine physicians with over 35 man-years of experience reporting MPI SPECT between them. Data were viewed in matching pairs of, in the first instance, the baseline and post-MI studies, and in the second case comparing the baseline and final imaging time-point to assess changes in perfusion, including a review of the gated images to assess wall motion abnormalities. A clinical report was generated for each pair of studies using the standard cardiac review format as recommended by the current guidelines for MPI reporting (22, 23).

**Figure 2:**
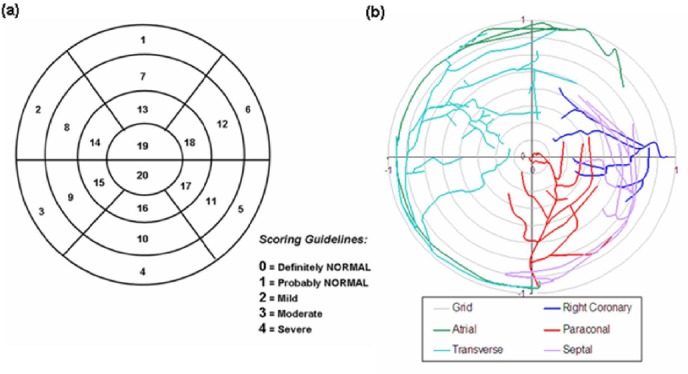
(a) The 20 segment map used by the expert reviewers for recording the perfusion defect location in both the post-MI and post-therapy studies, including a severity score per segment. (b) A polar map display representing the typical arterial coronary supply to the ovine heart, constructed by manual laser rotary scanning. This can be overlaid on the MPI polar map to aid in identification of the coronary supply to the damaged myocardium.

Each reviewer was provided with a polar map display template containing the 20 segment model overlay with a scoring guideline to record the location and severity of the abnormality (Figure 2). A categorical 5-point scoring scale was used ranging from 0 for normal through to 4 for a severe abnormality. The results were analysed using a Kappa (κ) statistic to measure agreement between reviewers (24).

Matching pairs of data (Baseline/Post-MI and Baseline/Post-therapy) were analysed using the PERfit Cardiac package (Hermes Medical, Nuclear Diagnostics, Sweden). Short axis slices were used to generate circumferential profiles from apex to base of heart that contained 12 profiles each with 40 elements, giving a total 480 elements per study (9° arcs). The mean change from baseline to post-MI and post-therapy for each element was calculated with a change of ≥ 10% being considered significant. The z-score of each element was calculated to quantify the number of standard deviations that the original value is relative to the mean and to determine change in perfusion from baseline.

#### Echocardiography Protocol

An echocardiogram was performed at baseline in all animals and after the intervention in a selected cohort of the animals. The sheep were positioned in the right decubitus position and secured to the imaging bed with the limbs extended forward. The sheep remained conscious during the procedure however a patch was placed over the eyes to reduce animal stress and movement. An area behind the left forelimb was shaved to ensure good contact of the transducer with the skin. Measures of LVEF, EDV and ESV were recorded to assess myocardial function and for comparison with gated MPI results.

#### Post Mortem Procedures

The sheep were euthanized after the final imaging time-point 6-8 weeks after the MI. Whilst anaesthetised, the sheep were injected with 400 MBq of [^99m^Tc]-Sestamibi so as to allow imaging of the heart for visual confirmation of the infarct location with the *ex-vivo* anatomic slices. After 5 minutes the sheep were sacrificed without recovery from anaesthesia using a lethal dose (2 g I.V.) of potassium chloride (KCl) given intravenously via the indwelling venous catheter. After the heart ceased beating, body tissues including the heart, lung, liver and kidneys were excised for further examination. The heart was fixed with formalin and then sectioned evenly through the short axis plane into 5 to 10 mm thick slices for histological sampling. The heart slices were placed on a template ordered from apex to base and imaged by placing the template directly on top of the collimator of an upturned gamma camera using a 512 × 512 matrix for 300 seconds. The heart sections were reviewed microscopic-ally to confirm the presence of an MI, to assess the extent, if any, of neovascularisation, and for correlation with MPI findings.

### Results

The study enrolled 18 ewes ranging in weight from 45 to 50 kg with a baseline resting LV EDV of 80 to 90 ml measured by echocardiography. Ten sheep completed the full protocol including *post mortem* anatomical correlation. Within the three treatment groups there was a control group (n=3), an intra-coronary infusion group (n=3) and an intra-myocardial injection group (n=4).

During the MI induction procedure six sheep had irreversible ventricular fibrillation and succumbed. Of the remaining 12 animals, sheep #8 had a perfusion abnormality at baseline imaging in the apical region and was excluded.

Sheep #9 successfully completed baseline imaging and proceeded to MI induction, however, no subsequent perfusion defect was seen on the MPI study and was therefore excluded. The results for all animals are summarised in Table 1.

**Table 1:** Summary of Results for all Sheep

Sheep No	Intervention	EDV (ml)			ESV (ml)			Gated MPI LVEF(%)			Echo LVEF(%)		Perfusioi Loss§ PMI(%)	Perfusion Loss§ PI(%)	MPI SPECT Clinical Interpretation Perfusion Change	Report from Anatomical Pathology
		Base	PMI	PI	Base	PMI	PI	Base	PMI	PI	Base	PI				
**S001**	†	54	*	*	19	*	*	64	*	*		*	*	*		
**S002**	C	45	52	40	15	21	20	67	59	50	54.4		14	16	Increased defect size segment 5	**Infarct confirmed**
**S003**	C	37	40	41	14	15	20	63	60	52	46.7		23	23	No change	**Infarct confirmed**
**S004**	IM	61	62	57	20	30	26	66	52	53	49.1		17	17	No change	**No significant neovascularisation**
**S005**	IM	69	71	70	24	34	31	65	46	55	62.2		26	18	Improvement periphery segments 13-15	**‘striking’ neovascularisatiion**
**S006**	†	53			22			59				*	*	*		
**S007**	IC	50	57	57	22	29	29	56	49	53	57.2	**	18	16	No change	**Nu significant neovascularisation**
**S008**	R	61			34			47				*			-	**-**
**S009**	R	59	66		27	35		54	47			*			-	**-**
**S010**	†	72			48			32				*			-	**-**
**S011**	IC	44	60	50	15	29	20	62	53	59	49.2	**	31	14	Improvement seen segments 13,19,20	**‘striking’ neovascularisatiion**
**S012**	C	37	74	58	16	40	29	56	46	47	41.1	**	17	22	No change	**Infarct confirmed**
**S013**	†	66			41			34							-	**-**
**S014**	IM	64	87	83	27	49	43	58	43	48	57	49.2	28	26	No significant change	**Zones of early neovascularisation**
**S015**	†	48			25			49				*				
**S016**	IM	47	55	53	23	29	33	51	47	42	45	46.7	29	17	Improvement seen segments 8.9.15-17,19,20	**Mild lymphohistiocytic inflammation & some vascularity**
**S017**	†	47			25			47							-	**-**
**S018**	IC	47	57	64	25	29	39	47	49	38	**	54.4	42	48	Increase defect severity segments 13,16,17.19. 20	**Infarct confirmed with lymphocytic inflammation**
***Overall Mean***		*53.4*	*61.9*	*57.3*	*24.6*	*30.9*	*28.8*	*54.3*	*50.1*	*49.7*	*51.3*	*50.1*	*24.5*	*21.7*		
***Overall SD***		*10.5*	*12.5*	*12.9*	*8.9*	*9.0*	*80*	*10.3*	*5.4*	*6.2*	*6.8*	*3.9*	*8.5*	*10.0*		
***Control Mean***		*39.7*	*55.3*	*46.3*	*15*	*25.3*	*23*	*62*	*55*	*49.7*	*47.4*	*··*	*18*	*20.3*		
***IC Mean***		*47*	*58*	*57*	*20.1*	*29*	*28.7*	*55*	*50.3*	*50*	*52.3*	*54.4*	*30.3*	*26*		
***IM Mean***		*60.3*	*68.8*	*65.8*	*23.5*	*35.5*	*33.3*	*60*	*49.5*	*49.5*	*53.3*	*48*	*25*	*19.5*		

† Deceased during infarct procedure; C = Control; R = removed from study; Base = Baseline; PMI = Post-Myocardial Infarction; PI = Post-Therapy; IM = Intramyocardial Injection; IC = Intracoronary Infusion;

* Discontinued in the study

** Echocardiography not performed

§ Perfusion loss from baseline study

#### Clinical Interpretation

**Figure 3:**
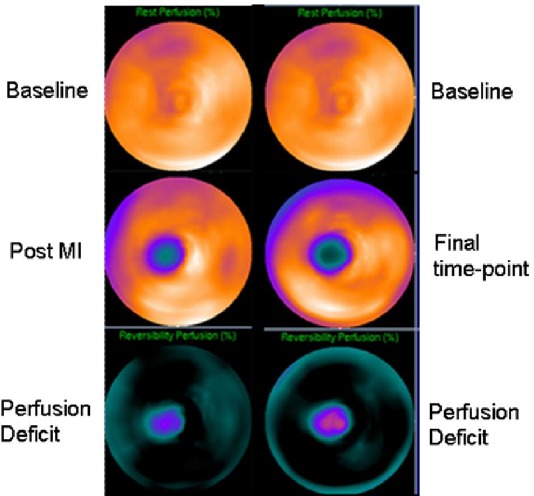
A comparison of the polar map perfusion change from a single baseline to post-MI (left) and final time-point (right) for sheep #12 indicating an increase in the size and extent of the perfusion defect between studies.

All images from the final cohort of sheep that completed the full imaging protocol were suitable for reconstruction and analysis using the conventional software tools available for clinical interpretation of MPI studies in man. In the control arm, no improvement in perfusion was identified from the post-MI images to the final imaging time-point. An example is shown in Figure 3 (sheep #12), where the baseline study shows normal uniform tracer distribution throughout the myocardium with a subsequent perfusion deficit seen post-infarction in the apex with a change in perfusion of -17%. The defect size increased at the final time-point with a further loss in perfusion to -22% relative to baseline. This was correlated microscopically with identification of fibrous scar tissue with lymphoid aggregates, histiocytes and calcium deposits in the same region. The gated MPI LVEF showed a decline of -14% from baseline to post-therapy (Table 1).

#### Quantitative Analyses

**Figure 4:**
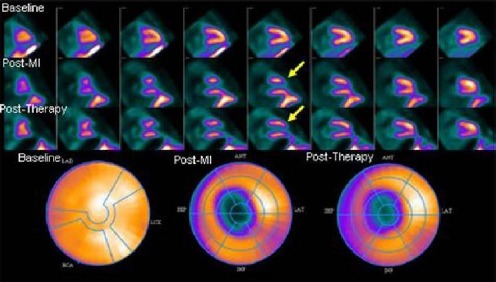
Comparison of the change in perfusion from baseline to post-MI and baseline to post-therapy for sheep #11 showing recovery of perfusion in the periphery of the infarct zone (yellow arrows). The vertical long axis (VLA) images show improvement in perfusion at the border zone of the infarct site on the post-therapy images as compared to the post-MI data.

Overall, in this small cohort the mean LVEF for both the IC and IM MSC implantation methods showed no statistically significant improvement in LV function. An average change of -7% and 5.3% in LVEF from baseline to post-MI and post-therapy respectively was seen in all the control sheep. A slight but not statistically significant improvement in LVEF was seen in 3 of 4 studies following IM implantation and in 2 of 3 following IC implantation. However, three of the four sheep using the IM method and one of the three using the IC method had a measurable improvement in perfusion. An example is shown in Figure 4. Sheep #11 had a significant perfusion deficit at the apex with a change of -31% that improved to -14% post-therapy, with a corresponding increase in LVEF of 6% from immediate post-MI. The images are suggestive of perfusion recovery in the border zones of the infarct site.

The overall agreement between the reviewers for all segmental scores was found to be 0.945 (with 1.0 being total agreement), calculated by dividing the matching agreements by the total segments (378/400). Quantitative agreement between the reviewers was determined by using the kappa statistic (К) with the results contained in Table 2. The calculation of К for this study is shown in Appendix I. The agreement in this study was К=0.89 which is regarded as very high agreement (24).

**Table 2:** Segmental scores by blinded reviewers of the post-MI and post-therapy images.

		Reader A					
**Severity**	**0**	**1**	**2**	**3**	**4**	*Total*
*Reader B*
	0	258	0	5	0	0	*263*
	1	3	4	0	0	0	7
	2	3	0	38	0	0	41
	3	3	0	1	42	3	49
	4	3	0	0	1	36	40
	*Total*	270	4	44	43	39	*400*

Scoring criteria where 0= Definitely Normal, 1 = Probably Normal, 2 = Mild, 3 = Moderate and 4 = Severe.

#### Using Pilot Data Results to Design Future Trials

The significance of change was assessed using a semi-quantitative scoring algorithm (QPS software) with a change in perfusion of >10% considered significant. Based on data already available for assessing LV function using either gated blood pool imaging or echocardiography, perfusion recovery from post-MI to post-therapy of ≥ 5% could be considered a significant change (25-27).

**Figure 5:**
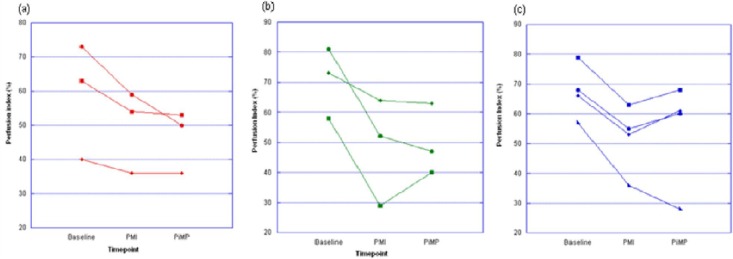
The mean change in perfusion from baseline to post-MI (PMI) in the affected segments was in the range of 6% to 82%/Following intervention (a) the control arm (red) showed no significant improvement in perfusion, however in (b) 1 of 3 intracoronary group (green) and (c)3 of the 4 in the intramyocardial group (blue) showed mild to moderate perfusion recovery.

The mean change in perfusion from baseline to post-MI was calculated as -22%, with a standard deviation of ±10.1% (Figure 5). To calculate the number of animals needed in any future study based on a change from baseline of -22% (*d*) with a standard deviation of ±10 (*s*), the standard difference was therefore equal to ~2 (*d/s* = 22/10). Using a study power of 0.8, the number of animals required to demonstrate a change due to the MI would be 8, and with a study power of 0.9, this number would increase to 12.

Assessing the recovery after therapy was calculated based on a minimum change of 7% and a maximum change of 12%. The standard difference for a 7% change and standard deviation of ±10 is 0.7 and for a 12% change it is 1.2. Therefore the number of subjects needed to detect a 7% change with a study power of 0.8 would be 60 and for a power of 0.9 it would be 80 animals. However, if the change in perfusion was greater, e.g. 12%, the number of animals required becomes 22 (for 0.8) and 28 (0.9) respectively.

The previous methods described to assess perfusion recovery following therapy were based on mean segment values generated by the QPS software package. However, as myocardial repair is more likely to occur around the edges of the infarcted tissue, a voxel level analysis using PerFit was undertaken. Results would suggest that two of the four sheep in the IM treatment group (sheep #5 and #14) have shown an improvement in perfusion in the infarcted area following stem cell therapy as well as an increase in global LVEF of 9% and 6% respectively. The perfusion score and infarct zone results show a positive shift in the perfusion scores in the infarct zone with the z-score values indicating a decrease in variability to those areas. This correlates with the anatomical findings of prominent and early neovascularisation in the same region. Of the three sheep in the IC arm, two of three sheep (sheep #7 and #11) had an improvement in perfusion following MSC implantation, correlating with an increase in global LVEF of 4% and 6% respectively.

**Figure 6:**
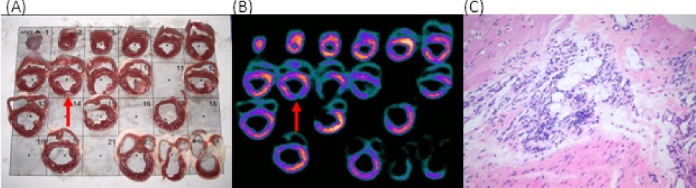
Sheep #3 site of MI induction as indicated by (A) the presence of fibrous scar tissue on the anatomical sheep heart slices correlating with (B) a perfusion defect seen in the MPI heart slices (red arrow). (C) The presence of lymphoid aggregates microscopically indicates the formation of scar tissue.

Confirmation of an area of infarcted tissue was seen microscopically for all studies by identification of fibrous scar tissue with the presence of lymphoid aggregates, histiocytes and calcium deposits indicative of myocardial injury (Figure 6C). Recovery of perfusion and function at the periphery of the infarct zone was seen in two of four IM and two of three IC studies that correlated with anatomic findings of early or prominent neovascularisation. The presence and site of the acute myocardial infarction identified microscopically correlated with the location of a perfusion defect on the *ex-vivo* [^99m^Tc]-Sestamibi heart slices in 5 of the 7 sheep (Table 3).

**Table 3:** Visual correlation of the presence and location of a perfusion loss on the [99mTc]-sestamibi heart slices and scar tissue on the anatomical heart slices.

Sheep #	[^99m^Tc]-Sestamibi slices	Anatomical slices	Visual Correlation
**3**	Lateral wall; on 3 slices	Lateral wall white scar tissue on matching 3 slices	Y
**4**	Latero-apical defect; on 3 slices	Latero-apical pinkish vascularity on matching 3 slices	Y
**9**	No perfusion abnormality; correlates with MPI finding	Lateral wall pinkish vascularity on 2 slices	N (small defect microscopically)
**11**	Apical perfusion defect on 4 slices only	Apical white scar tissue on 3 slices; pinkish vascularity on 3 slices	Y
**12**	Apico-septal perfusion defect on 4 slices	Apico-septal white scar tissue on matching 4 slices	Y
**16**	No perfusion deficit identified	Heart slices thick and uneven; cannot identify infarct	N
**18**	Large apico-septal perfusion defect on 4 slices	Large apico-septal white scar tissue on matching 3 slices	Y

## Discussion

The main aim of this study was to evaluate whether standard nuclear medicine gamma cameras, radiopharmaceuticals, software analys-es and reviewing workstations could be used to assess a large animal model of recovery from myocardial infarction, in this case, after therapy with stem cells delivered by a variety of routes of administration. After minor adjustments to account for the different orientation of the heart in the sheep as compared to man we have found that the same techniques can be employed. This now provides a platform to study aspects of MSC therapy that have previously required specialised small animal scanning equipment (e.g., for mice and rats), precluding many institutions such as our own. Our experiences suggest that this model could be reproduced in many institutions with nuclear medicine departments, and thus wide-spread experience with a realistic model of acute

myocardial infarction and response to therapy could be accelerated with many more centres participating. We used a SPECT/CT hybrid scanner to give accurate scatter and attenuation corrected SPECT images and such devices are now ubiquitous in the practice of nuclear medicine. The use of CT-based attenuation correction is now recommended, and common, in many nuclear medicine practices. Scatter correction further improves the quality and accuracy of the data.

Animal models and imaging have been used extensively to assess and trial new treatments, novel radiotracers and improved targeting techniques prior to their use in humans. While some animal models closely resemble the anatomical and physiological structure of humans, large differences are often seen between small and large animal models. The similarity of the mouse to the human genome is attractive to researchers as it allows for genetic manipulations to be performed in the mouse that closely simulate human disease (28), however, differences in cardiac size, anatomy and molecular machinery are significant, especially in longitudinal imaging studies such as this one. Therefore, large animal models such as the canine, porcine and ovine species have been used to evaluate cardiac disease due to their closer similarity to the human heart. Historically, the canine model has been used to examine the effects of chronic myocardial ischemia and myocardial infarction on LV function, geometry and structure (29). While dogs have a significant degree of collateral circulation which make it difficult to generate consistent myocardial injuries (30), the porcine and ovine heart exhibits coronary artery supply and gross anatomic structure similar to that of humans (31, 32). A quantitative study by Weaver *et al* evaluated the anatomy and distribution of porcine coronary arteries in which they compared their findings with those reported for canines and humans (33). The existence of ‘scant coronary collaterals’ in pigs was reported primarily localized to the mid myocardium and sub-endocardium. Studies have also shown that the ovine model of myocardial ischemia does not exhibit significant collateral coronary arteries with essentially separate coronary circulations to the two ventricles (34). Both are important advantages of this model for use in metabolic and mechanical functional studies of acute MI (35). Coronary artery ligation in sheep produces the pathological, haemodyna-mic and neurohormonal characteristics of compensated LV impairment secondary to MI. This is a reproducible model that reflects the clinical condition and therefore can be used to investigate the pathophysiology of early LV dysfunction as well as experimental therapies (36).

The size of the sheep heart is similar to a small female with an EDV of approximately 80 ml and a normal LVEF of greater than 50%, similar to that of a healthy human thus making it amenable to imaging with current SPECT gamma cameras. As a consequence of the small size, however, difficulties may arise in accurately tracking the endocardial and epicardial borders on the gated MPI studies resulting in uncertainties in the LVEF values obtained. Alternately, the measurement of LVEF using gated heart pool scanning with ^99m^Tc-labelled erythrocytes (RBCs) in planar and/or SPECT modes is known to produce reliable and reproducible measures of LV function in standard clinical practice in man (37) that is less affected by the spatial resolution limitations of the gamma camera. The addition of this approach to the existing study protocol should reduce errors in assessing LV function and will therefore be included as part of the standard imaging protocol for any future studies.

Our experienced nuclear medicine physicians found no difficulties in interpreting the MP images derived. This was confirmed by a К value of 0.89 indicating very good agreement. Total agreement gives a score of 1, with 0 indicating no agreement while negative values mean worse than by chance. A К value between 0.81-1.0 is said to represent very good agreement (38).

A pilot study enables the investigator to test a proof of concept, with the benefit being its value in assessing the quality of the data, checking the logistics of the trial and enhancing reliability of estimates for use in sample size calculations. The logistics of testing such methodologies in human trials is complex and hard to undertake in a controlled manner hence most are performed using animal modelling. The results reported from this pilot study have demonstrated that standard clinical imaging techniques and investigations used to assess myocardial injury and response to therapy in man can be used in large animal models to test response to novel treatments such as the use of stems cells for myocardial repair. This pilot study only included small numbers of animals in each treatment group and therefore determining the most effective implantation method to improve both perfusion and function following an induced MI may not be possible. However, these initial results would suggest that both the IM and IC methods could potentially be used for this type of therapy, with the IM method in this pilot study showing an improvement in LV function and neovascularisation identified microscopically. Additionally, the experience gained with these techniques can be easily translated to human studies to assess therapeutic benefit in the clinical setting.

## Conclusions

The anatomy and coronary arterial supply of the sheep heart is similar to the human heart with very little collateral supply, and an infarct can be reliably and reproducibly induced making it an ideal model for testing therapeutic interventions post-MI. The decision to study the ‘functional consequences’ rather than directly labeling the stem cells to study the fate of the MSCs *in vivo* was deliberate as this process has proven to be a reliable and reproducible method of assessing response to treatment which can be readily translated to human studies. This pilot study has shown that techniques used in the clinical setting in man, which have been highly developed and refined over many years and hundreds of thousands of investigations, can be used with large animals to follow the consequences of stem cell and other therapies. Also, this study has shown that large animal models are suitable for assessment with standard myocardial perfusion imaging, radiopharmaceu-ticals and protocols which could subsequently be applied in human clinical trials to study the efficacy of stem cell therapy. As such, the sheep model and the nuclear medicine imaging and analysis techniques used here have demonst-rated an excellent translational platform for bridging ‘mouse-bound’ pre-clinical studies and eventual clinical therapeutic use in humans. Further, both qualitative and semi-quantitative analysis can be applied to allow repeated non-invasive temporal assessment of extent, severity, defect size and LV function using standard cardiac analysis software and review packages available in the majority of nuclear medicine departments.

## Appendix I

### Calculation of the Kappa Statistic

The possibility of agreement by chance alone needs to be considered and this is done by calculating the expected frequencies for each severity score from Table 2 as below:

(0) Definitely Normal 263×270/400=177.5
(1) Probably Norma l7×4/400=0.07
(2) Mild 41×44/400=4.51
(3) Moderate 49×43/400=5.27
(4) Severe 40×39/400=3.9

Therefore, the total number of agreements expected by chance are 190.8, with the proportion of the total being 190.8/400 = 0.47. The К is then:
